# Effect of TNF inhibitors on arterial stiffness and intima media thickness in rheumatoid arthritis: a systematic review and meta-analysis

**DOI:** 10.1007/s10067-023-06505-y

**Published:** 2023-01-16

**Authors:** Bafrin Abdulmajid, Annelies B. Blanken, Eva H. van Geel, Joost G. Daams, Michael T. Nurmohamed

**Affiliations:** 1 Department of Rheumatology, Amsterdam Rheumatology and Immunology Center, Location Reade, Amsterdam Dr. Jan van Breemenstraat 2, PO Box 58271, 1040 HG Amsterdam, The Netherlands; 2 Amsterdam UMC, Location AMC, Amsterdam, The Netherlands; 3 Department of Rheumatology, Amsterdam Rheumatology and Immunology Center, Location Amsterdam UMC, VU University Medical Center, Amsterdam, The Netherlands; 4 Department of Medical Library, Amsterdam UMC, Location AMC, Amsterdam, The Netherlands

**Keywords:** Cardiovascular burden, Rheumatoid arthritis, Systematic review, Tumor necrosis factor inhibitors

## Abstract

Controlling inflammation with tumor necrosis factor (TNF) inhibitors in rheumatoid arthritis (RA) patients is hypothesized to reduce their cardiovascular risk. We performed a systematic review and meta-analysis on the effects of TNF inhibitors on arterial stiffness and carotid intima media thickness (IMT) in RA. MEDLINE, EMBASE, clinicaltrials.gov, and WHO Clinical Trials Registry were searched up to September 2021 for randomized controlled trials, prospective cohort studies, and nonrandomized clinical trials evaluating the effects of TNF inhibitors on pulse wave velocity (PWV), augmentation index (AIx), and IMT in RA. A meta-analysis was performed to assess changes of these measures after therapy during different follow-up periods. Risk of bias assessment was performed using an adjusted Downs and Black checklist (INPLASY: 2022-1-0131). Thirty studies were identified from 1436 records, of which 23 were included in the meta-analysis. PWV and AIx showed a decrease after treatment (PWV: mean difference (MD) −0.51 m/s (95% CI: −0.96, −0.06), *p*=0.027; AIx: MD −0.57% (95% CI: −2.11, 0.96), *p*=0.463, sensitivity analysis AIx: MD −1.21% (95% CI: −2.60, 0.19), *p*=0.089). For IMT, there was a slight increase in the first months of follow-up, but this disappeared on the long-term (overall timepoints MD −0.01 mm (95% CI: −0.04, 0.02), *p*=0.615). Heterogeneity was high in the overall analyses and subgroups with long follow-up periods (≥12 months). The included studies showed mixed results of the effects of TNF inhibitors on the surrogate markers. The pooled results suggest that PWV and AIx decrease over time, while IMT remains stable. This indicates a favorable effect of TNF inhibitors on the cardiovascular disease risk, all the more since these markers also increase with age.

**Supplementary Information:**

The online version contains supplementary material available at 10.1007/s10067-023-06505-y.

## Introduction

Rheumatoid arthritis (RA) is associated with an increased risk of cardiovascular disease (CVD) and CVD-associated mortality [1–3]. The mortality, adjusted for age and sex, is 0.5–2 times higher in RA patients compared to healthy individuals [4], and cardiovascular diseases are responsible for about 50–60% of this mortality in RA patients [5]. Furthermore, these patients have a 50% higher chance of a cardiovascular event [6]. It is known that RA patients have increased prevalences of traditional cardiovascular risk factors (e.g., hypertension, dyslipidemia, and diabetes) [7]. However, the chance of cardiovascular events of RA patients is higher than expected based on the prevalence of these traditional cardiovascular risk factors alone [8]. This suggests that chronic inflammation itself contributes to (accelerated) atherosclerotic plaque development and instability [1–3]. Studies suggest that the increased cardiovascular risk can be reduced by controlling inflammation [9]. In particular, tumor necrosis factor (TNF) inhibitors appear to have a favorable effect on patients’ cardiovascular risk [5]. The hypothesis is that this is due to directly reducing inflammation in the arterial wall [9]. Surrogate endpoints can be used to determine the effects of TNF inhibitors on subclinical atherosclerosis in a noninvasive manner [10]. Arterial stiffness (pulse wave velocity (PWV), augmentation index (AIx)) and arterial wall thickness (carotid intima media thickness (IMT)) are well-established markers for subclinical cardiovascular disease. These markers give insight into the progress of atherosclerosis and are therefore determinants of the cardiovascular risk in RA patients [11].

The last decade the cardiovascular burden of RA patients has attracted more attention from researchers. Several studies have examined the effects of TNF inhibitors on the increased cardiovascular risk in RA patients. However, a recent overview of these studies investigating the effect of these agents particularly on RA patients is still lacking [12]. Evaluating these surrogate markers will provide insight whether a commonly used treatment in RA patients also has a beneficial effect on cardiovascular disease. Therefore, the aim of this systematic review and meta-analysis is to evaluate the effect of TNF inhibitors on arterial stiffness (as measured with PWV and AIx) and IMT in RA patients.

## Methods

This systematic review was reported in accordance with the Preferred Reporting Items for Systematic Reviews and Meta-Analyses (PRISMA) checklist (Supplementary Data S1). This protocol was registered in the International Platform of Registered Systematic Reviews and Meta-analysis Protocols (INPLASY), on 28 February 2022 (registration number: 2022-1-0131)

### Eligibility criteria

Studies were included if (1) the population analyzed was diagnosed with RA, or at least 80% of the study population consisted of RA patients, (2) the intervention was a TNF inhibitor or any combination of drugs including an TNF inhibitor, and (3) the outcome measure was either PWV, AIx, and/or IMT. Dosage and duration of the agents were not considered for inclusion. Patients had to start with the intervention directly after the baseline visit, or in case of follow-up ≥12 months no later than 3 months after the baseline visit. At least 80% of the patients needed to receive TNF inhibiting treatment. Only randomized controlled trials (RCTs), prospective cohort studies, and nonrandomized clinical trials were included. Studies not providing longitudinal data (pre-post treatment values or change over time) of the outcome were excluded. For a sensitive search, there were no restrictions based on the language or publication date.

### Search strategy and study selection

**Fig. 1:**
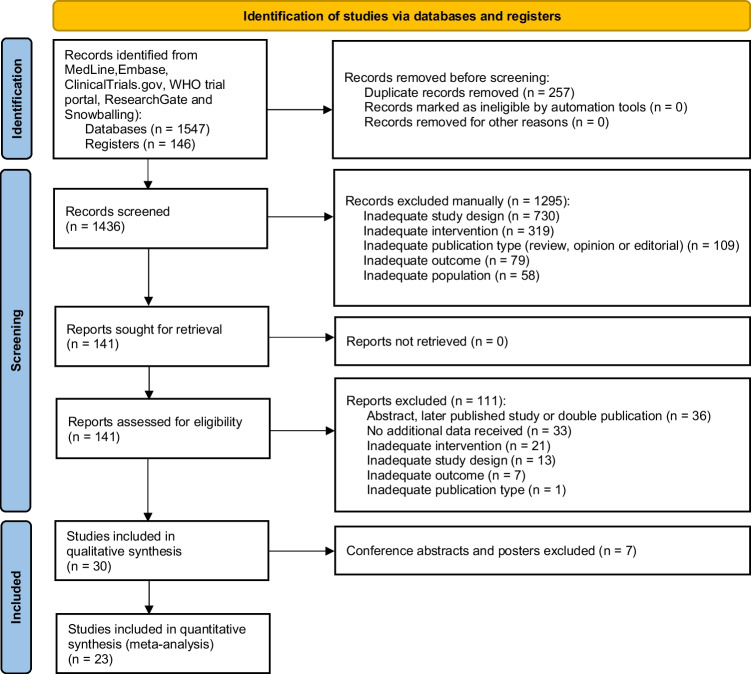
Prisma study selection flowchart [13]

We searched MEDLINE, EMBASE, clinicaltrials.gov, and WHO international Clinical Trials registry from inception until 15 September 2020 (Fig. 1 and Supplementary Data S2). Furthermore, similarity tracking for eligible studies, a citation search and screening of reference lists were used to identify relevant articles. ResearchGate profiles of top authors on the subject were investigated to identify relevant data points. When necessary, study authors were contacted by e-mail for additional or missing data. Two independent reviewers (A. B. and B. A.) screened the articles and abstracts using the Rayyan QCRI screening tool [14]. Disagreements were solved by reviewing full texts when available and discussed until consensus was reached. Subsequently, full papers were obtained for detailed assessment by the two independent reviewers (A. B. and B. A.).

### Data extraction

Primary outcome measures for this systematic review were the effects of TNF inhibiting treatment on IMT, PWV, and AIx. For this purpose, one reviewer (A. B. and B. A.) collected the following data with the use of a standardized data extraction form, such as study characteristics, outcome measure at baseline, follow-up and/or MD, and measurement method (Supplementary Data S3). The principal study measures were baseline, follow-up, and MD of PWV, AIx, and IMT after TNF inhibiting treatment. A second reviewer (A. B., B. A., or E. G.) checked all data independently, and any differences were discussed. In studies providing data only in figures, the numbers were extracted with the WebPlotDigitizer tool [15] by two authors (A. B. and B. A.), and the mean of the two measurements were used. Additional data received via contacting study authors were checked by two independent reviewers (A. B. and E. G.).

### Risk of bias assessment

Quality and risk of bias of all studies were assessed using an adjusted version of the Downs & Black checklist, by two authors (B. A. and E. G.) independently. This checklist consisted of 23 questions; a score between 0 and 23 could be obtained (Supplementary Data S4). A score of 16 or higher was considered good quality and a score under 12 as high risk of bias and poor quality. These studies were not included in the meta-analysis. In case of discrepancies, differences were discussed until consensus was reached, and whenever necessary, a third author (A. B.) was consulted. Quality assessment was performed on the study level, and in case when additional data were retrieved, quality assessment was only based on data available in the published article or abstract.

### Data synthesis

For the qualitative synthesis, the findings of the included studies were narratively described, grouped on outcome measure. Both the qualitative and quantitative syntheses (meta-analysis) focused on the effect of TNF inhibiting therapy over time within the same group of patients, as we expected a wide range of variance in the anti-inflammatory treatment used in the control groups. In addition, the anti-inflammatory treatment in the control group was expected to influence the vascular wall and cardiovascular risk in the same way as TNF-inhibitors, probably leading to no difference (or small differences) between the groups [16–18]. Therefore, studies were included in the meta-analysis when pre- and posttreatment measurements were performed and reported. Not reported MD and corresponding standard deviation (SD) were calculated using the pre- and posttreatment measurements and SDs. The results were stratified based on the primary outcome measure and follow-up period, because it was expected that the effect of TNF inhibiting treatment might be different for different treatment durations. The following follow-up periods were chosen to evenly distribute the studies along the groups: 0–2 months, 2–4 months, 5–12 months, and ≥12 months. For each outcome, an overall meta-analysis including all studies was performed. In case of multiple timepoints per study, only the longest follow-up time was included. Sensitivity analysis using the shortest follow-up was performed additionally. Subgroup summary estimates were only calculated when 3 or more studies were present.

A random effects model was used to compare the data. Heterogeneity was measured by *I*^2^ statistic, reflecting the percentage of total variance that can be explained by heterogeneity. Visual inspection of funnel plots was used to assess asymmetry, in addition to Egger’s test. The data were considered continuous outcomes and analyzed with MD (95% confidence interval (CI)). Statistical analysis was done in RStudio (version 3.6.1, using the metafor package [19]).

## Results

### Study selection

The database search yielded 1547 records and 146 records were identified through assessing trial registries, ResearchGate, and reversed snowballing of included studies (Fig. 1). After duplicates were removed, 1436 records were screened for eligibility based on title and abstract. A total of 1295 records were excluded, leaving 141 studies that were assessed for eligibility based on the full-text. Finally, 30 studies were included in qualitative synthesis and 23 in quantitative synthesis as 7 abstracts were excluded.

### Study characteristics

Six studies were RCTs, one was a posthoc analysis of an RCT and 23 were nonrandomized clinical trials or prospective cohort studies (Table 1) [20–49]. Seventeen studies had a control group. A combined total of 907 RA patients with a mean age of 54 (SD 10) years were examined. The follow-up duration of the studies ranged between 6 weeks and 4 years (interquartile range (IQR) 38 weeks). Of the 30 included studies, 14 reported PWV, 11 AIx, and 19 IMT.

**Table 1:** Overview of results of eligible studies on effect of TNF inhibitors on IMT, PWV, and AIx

Reference	Intervention	Results
Full-text articles		
Bergstrom, 2018 [20]	14 RA patients receiving adalimumab, no controls	No significant difference in IMT after 3 months
Daien, 2013 [21]	28 RA patients treated with etanercept, 20 controls receiving methotrexate, sulfasalazine, or leflunomide	No significant difference in PWV and AIx after 3 and 6 months of follow-up. No significant difference in the control group in PWV and AIx after 3 and 6 months of follow-up
Del Porto, 2007 [22]	30 RA patients treated with etanercept or infliximab, compared with 10 controls receiving no biologicals	Decrease in right and left IMT (*p*=0.0001) after 12 months of follow-up. No significant difference in the control group after 12 months
Di Micco, 2009 [23]	7 RA patients treated with infliximab, compared to 7 controls receiving no biologicals	Increase in IMT (*p*=0.026) after 12 months, no significant difference in the control group
Ferrante, 2009 [24]	40 RA patients receiving adalimumab, etanercept or infliximab after 3 months of methotrexate, compared to 79 controls receiving no biologicals	Decrease in IMT (*p*<0.0001) after 24 months, no significant difference in the control group
Galarraga, 2009 [25]	26 RA patients treated with infliximab, compared to 21 controls receiving methotrexate	Decrease in AIx@75 (*p*=0.025) after 2 and 4 months, no significant difference in the control group
Gonzalez-Juanatey, 2006 [26]	8 RA patients treated with infliximab, 15 controls treated with methotrexate, leflunomide or prednisone	No significant difference in IMT after 3 years of follow-up
Gonzalez-Juanatey, 2012 [27]	34 RA patients treated with adalimumab, no controls	No significant difference in IMT after 12 months of follow-up
Kerekes, 2011 [28]	8 RA patients treated with adalimumab, no controls	Decrease in IMT after 24 weeks (*p*=0.002), no significant difference in PWV
Komai, 2007 [29]	15 RA patients treated with infliximab, no controls	No significant difference in PWV after 2 and 6 weeks
Kume, 2011 [30]	41 patients treated with either etanercept or adalimumab, compared to 22 controls receiving tocilizumab	No significant difference in IMT after 24 weeks of follow-up, decrease in AIx@75 (etanercept (*p*=0.03), adalimumab (*p*=0.02)) after 24 weeks, no significant difference in the control group
Maki-Petaja, 2006 [31]	9 RA patients treated with etanercept, no controls	Decrease in aortic PWV (*p*=0.0003) and no significant difference in brachial PWV and AIx after 4 weeks and 12 weeks of follow-up
Maki-Petaja, 2012 [32]	17 RA patients treated with either etanercept or adalimumab, no controls	Decrease in aortic PWV (*p*=0.04) and brachial PWV (*p*=0.06) after 8 weeks of follow-up. No significant difference in AIx after 8 weeks
Pieringer, 2010 [33]	17 RA patients treated with infliximab, no controls	Increase in AIx (*p*=0.01) after 7 weeks of follow-up
Sidiropoulos, 2009 [34]	12 RA patients treated with either adalimumab or infliximab, 6 controls receiving methotrexate, sulfasalazine, or hydroxychloroquine	No significant difference in IMT after 3 months and 18 months of follow-up. No significant difference in the control group
Taguchi, 2012 [35]	6 RA patients treated with etanercept, no controls	No significant difference in PWV after 12 months of follow-up
Tam, 2012 [36]	20 RA patients treated with infliximab, 20 controls receiving methotrexate	Lower PWV in the MTX + IFX group, compared to the MTX alone group (*p*=0.044) after 6 months of follow-up. No significant difference in IMT and AIx
Turiel, 2010 [37]	10 RA patients treated with adalimumab, 10 controls receiving methotrexate	No significant difference in IMT in the RA group after 18 months and no significant difference in IMT between RA patients and controls
Van Doornum, 2005 [38]	14 RA patients treated with adalimumab, etanercept or infliximab, no controls	No significant difference in AIx after 6 weeks of follow-up
Vassilopoulos, 2014 [39]	18 RA patients treated with adalimumab, 18 controls receiving methotrexate	Decrease in PWV after 12 weeks (*p*=0.00006), no significant difference in AIx. No significant difference in the control group in PWV and AIx after 12 weeks of follow-up
Vegh, 2020 [40] ^a^	26 RA patients treated with either etanercept or certolizumab pegol, no controls	IMT showed an increase after 6 and 12 months of follow-up, PWV showed no significant difference
Wasko, 2014 [41]	290 RA patients treated with either 100 mg golimumab + placebo, 91 with 50 mg golimumab + methotrexate or 100 mg golimumab + methotrexate, 92 controls receiving placebo + methotrexate	Slight increase of IMT in the 100 mg golimumab + methotrexate after 24 weeks when compared to the placebo + methotrexate group (*p* =0.005), while no difference after 52 weeks of follow-up. No significant difference after 24 and 52 weeks of follow-up in the 50 mg golimumab + methotrexate group and 100 mg golimumab compared to placebo + methotrexate group
Wong, 2009 [42]	26 RA patients treated with infliximab, no controls	No significant difference in IMT and AIx after 24 and 56 weeks of follow-up. PWV showed no significant difference after 24 weeks and a decrease after 56 weeks of follow-up (*p*=0.004)
Abstracts		
Blanken, 2019 [43] ^a^	31 RA patients treated with adalimumab, 30 controls receiving methotrexate, sulfasalazine, and/or hydroxychloroquine	No significant difference in IMT and PWV after 6 months and 4 years of follow up. AIx@75 showed a significant decrease after 6 months and no difference after 4 years of follow-up. In the control group there was no significant difference in IMT and PWV after 6 and 18 months, AIx@75 showed a decrease after 6 months and 4 years of follow-up
Heinz, 2018 [44] ^a^	22 RA patients treated with etanercept, certolizumab pegol or adalimumab, no controls	No significant difference in IMT after 3 and 12 months of follow-up
Oakley, 2016 [45]	26 RA patients treated with adalimumab, 27 controls receiving a placebo	Decrease in PWV after 4 and 12 weeks of follow up in the adalimumab group and no significant difference after 1 and 24 weeks. Compared to the control group in the group with established RA there was a decrease after 4 and 12 weeks and no significant difference after 1 and 24 weeks. In the group with early RA there was a significant decrease after 4 and 12 weeks and no significant difference after 1 and 24 weeks
Puntmann, 2011 [46]	13 RA patients treated with TNF inhibitors	Decrease in aortic PWV after 3 months
Swierkot, 2019 [47]	38 RA patients treated with either etanercept or adalimumab, 22 healthy controls	Decrease in IMT after 12 months of follow up (*p*<0.05)
Zacariaz, 2014 [48]	10 RA patients treated with TNF inhibitors, 15 controls receiving DMARDs	No significant difference in IMT and PWV after 12 months, no difference in control group
Zhu, 2016 [49]	50 RA patients treated with etanercept, 50 controls receiving DMARDs	Decrease in IMT after 24 weeks (no *p* value reported)

*AIx* augmentation index, *DMARDs* disease modifying anti-rheumatic drugs, *IMT* intima media thickness, *mg* milligram, *PWV* pulse wave velocity, *RA* rheumatoid arthritis, *TNF* tumor necrosis factor

^a^Additional data received upon request

### Risk of bias assessment

**Fig. 2:**
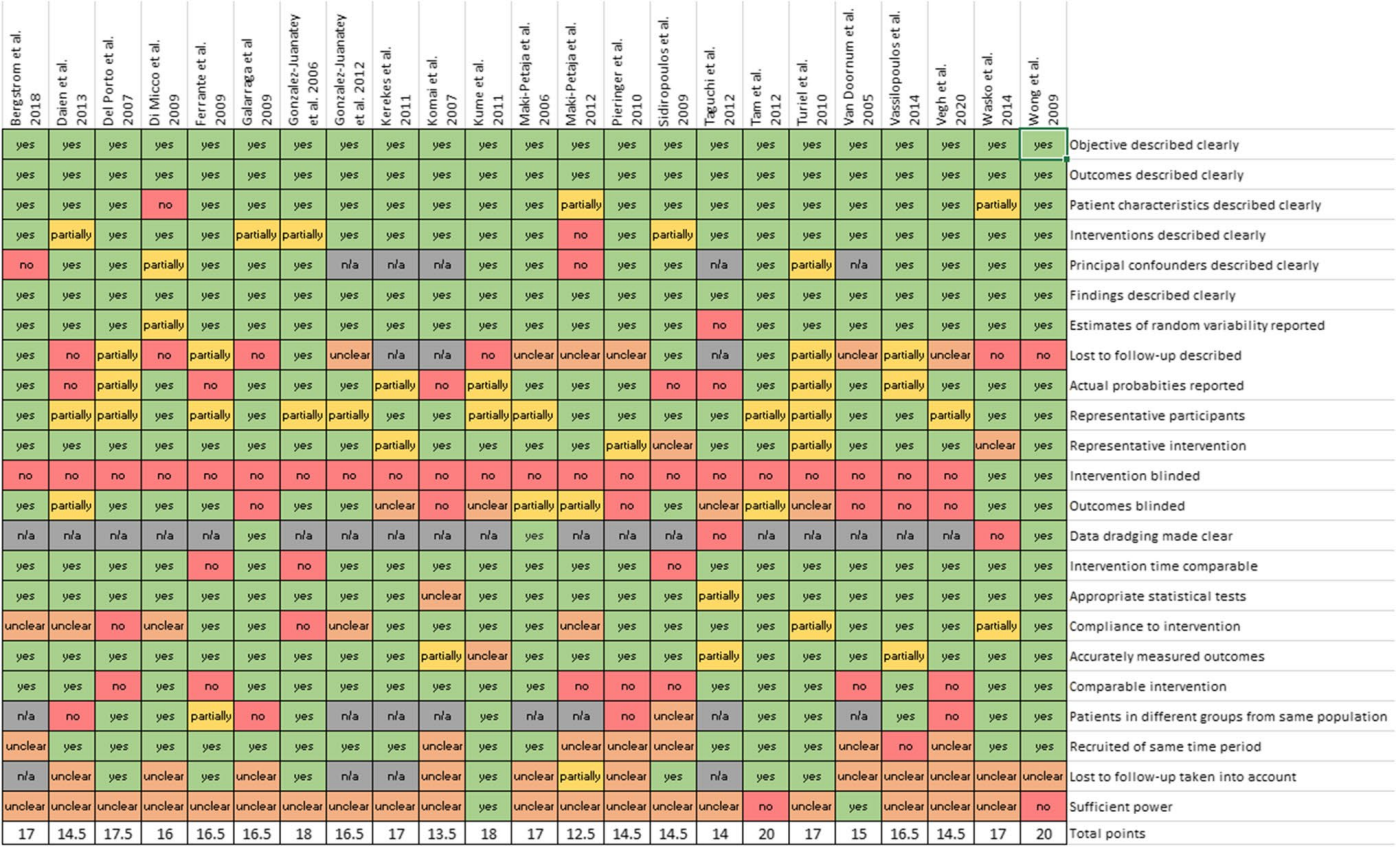
Overview of risk of bias assessment of peer-reviewed articles. Risk of bias assessment of abstracts is shown in Supplementary Figure 1 and full overview in Supplementary Data S4. Additional data or information that weas received via e-mail was not included in this assessment

An overview of the risk of bias assessment for peer-reviewed full-text articles is shown in Fig. 2 and in Supplementary Figure S1 for abstracts. In addition, complete data are included as Supplementary Data S5. Of the peer-reviewed articles, 15 studies were of good quality (≥16 points). The other studies were of lower quality, but scored at least more than 50% of total points (12.5–15). Poor performance in risk of bias was the result of methodology, blinding, insufficient reporting of losses to follow-up, and power. All abstracts had a high risk of bias (score 0–10 points), mostly due to lack of given information. Because of poor quality and high risk of bias, these studies were not included in the meta-analyses.

### Outcomes of studies

An overview of the results of the eligible studies is presented in Table 1. The complete data extraction table is included as Supplementary Data S3. Three studies responded to our request and provided additional data (Table 1).

### Pulse wave velocity

Eight studies measured the aortic PWV [21, 29, 39, 42, 43, 45, 46, 48], four studies the brachial PWV [28, 35, 36, 40], and two studies measured both [31, 32]. Seven studies showed a significant decrease in PWV: the studies of Maki-Petaja et al. 2006 [31] and 2012 [32], Oakley et al. [45], Puntmann et al. [46], Tam et al. [36], Vassilopoulos et al. [39], and Wong et al. [42]. A nonsignificant decrease was found in six studies: Blanken et al. [43], Daien et al. [21], Kerekes et al. [28], Taguchi et al. [35], Vegh et al. [40], and Zacariaz et al. [48]. The study of Komai et al. [29] showed a non-statistically significant increase.

### Augmentation index

Eleven studies reported AIx [21, 25, 30–33, 36, 38, 39, 42, 43], of which 4 studies reported the AIx corrected for heart rate (AIx@75) [25, 30, 36, 43]. All studies measured the aortic AIx, except for the study of Daien et al. [21] which measured the carotid AIx. Five studies [25, 30, 36, 39, 43] showed a decrease in AIx, which was significant in the studies of Galarraga et al. [25], Kume et al. [30], and Blanken et al. [43] in the first six months of follow-up. The remaining six studies [21, 31–33, 38, 42] showed an increase in AIx, of which one reached statistical significance (Pieringer et al. [33]).

### Intima media thickness

All studies reported the IMT of the common carotid arteries, of which one reported the mean IMT of the common carotid arteries and the carotid bulb [23] and two studies reported the mean IMT of the common and internal carotid arteries, and the carotid bulb [22, 36]. Of the 19 studies presenting IMT, seven studies [22, 24, 27, 28, 37, 47, 49] showed a decrease in IMT in the follow-up period. This decrease was statistically significant in the studies of Del Porto et al. [22], Ferrante et al. [24], Kerekes et al. [28], and Swierkot et al. [47]. Six studies showed an increase in IMT [20, 23, 26, 40, 41, 44, 48], which was statistically significant in three studies (Di Micco et al. [23], Vegh et al. [40], and Wasko et al. [41]). The studies of Blanken et al. [43], Kume et al. [30], Sidiropoulos et al. [34], Tam et al. [36], and Wong et al. [42] showed no change in IMT after the follow-up duration.

### Meta-analysis

**Fig. 3:**
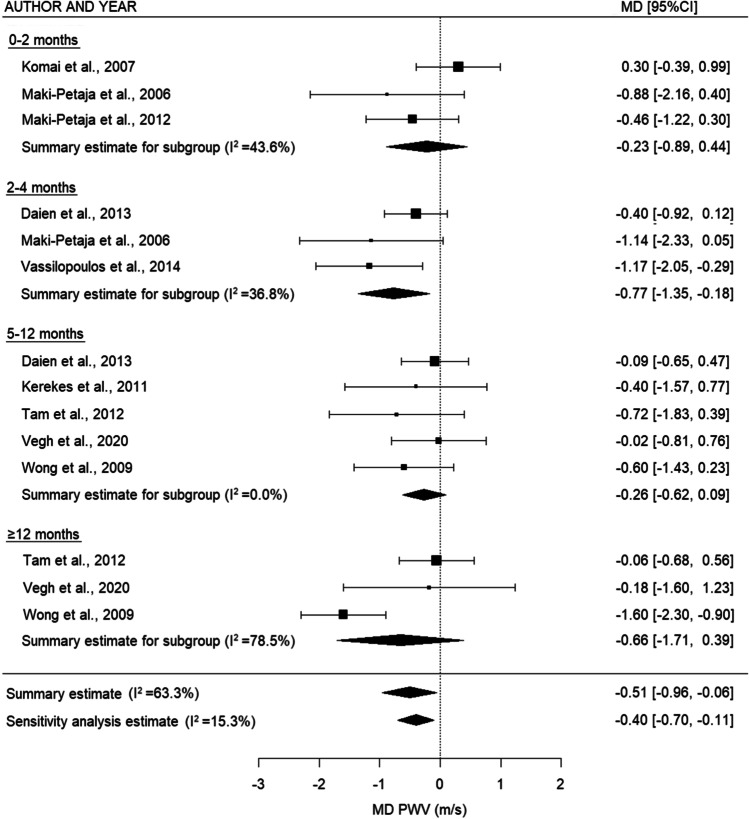
Forest plot for PWV comparing different time intervals. PWV: pulse wave velocity, MD: mean difference, m/s: meter/second, 95% CI: 95% confidence interval

**Fig. 4:**
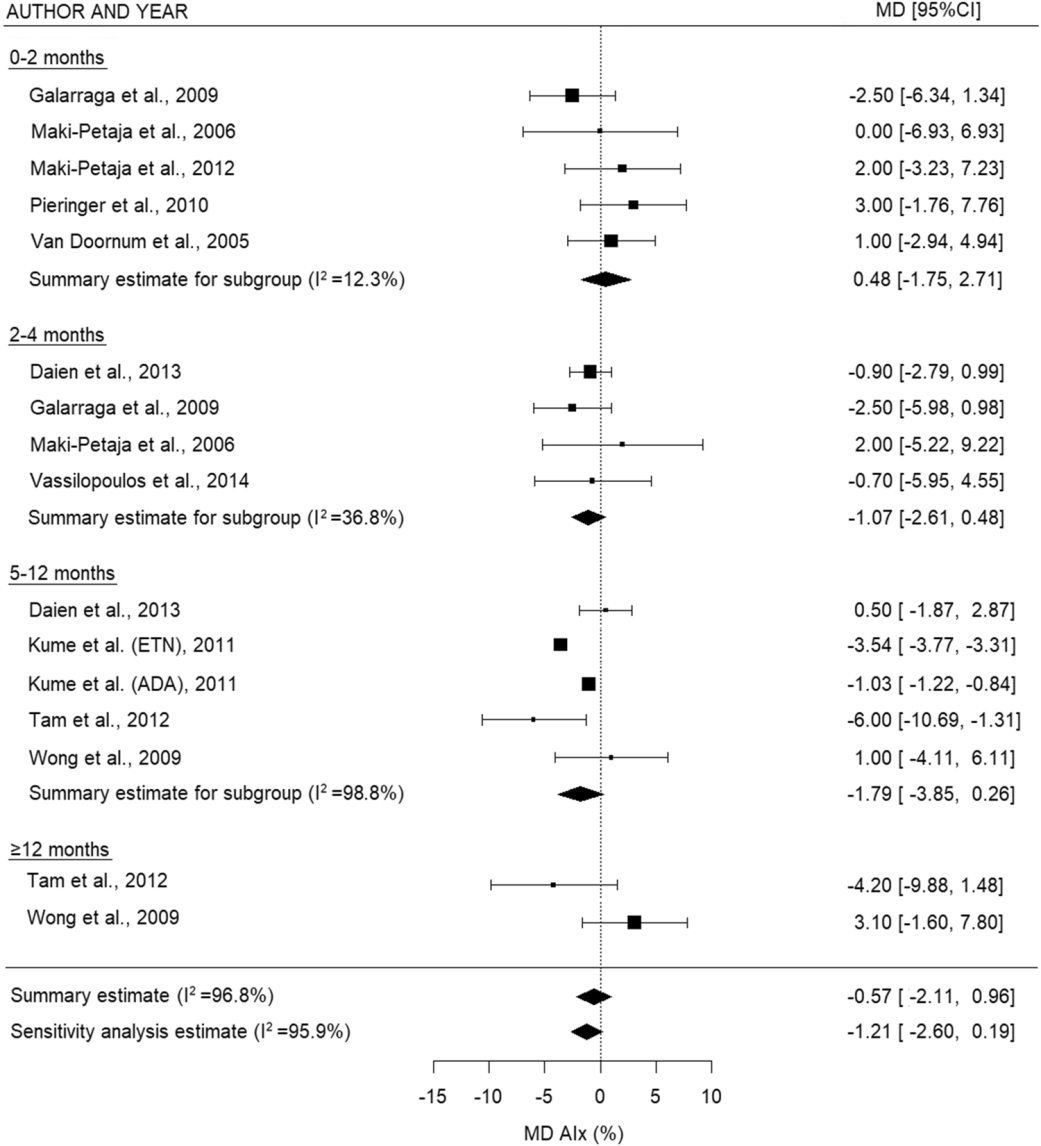
Forest plot for AIx comparing different time intervals. ADA: adalimumab, AIx: augmentation index, ETN: etanercept, MD: mean difference, 95% CI: 95% confidence interval

**Fig. 5:**
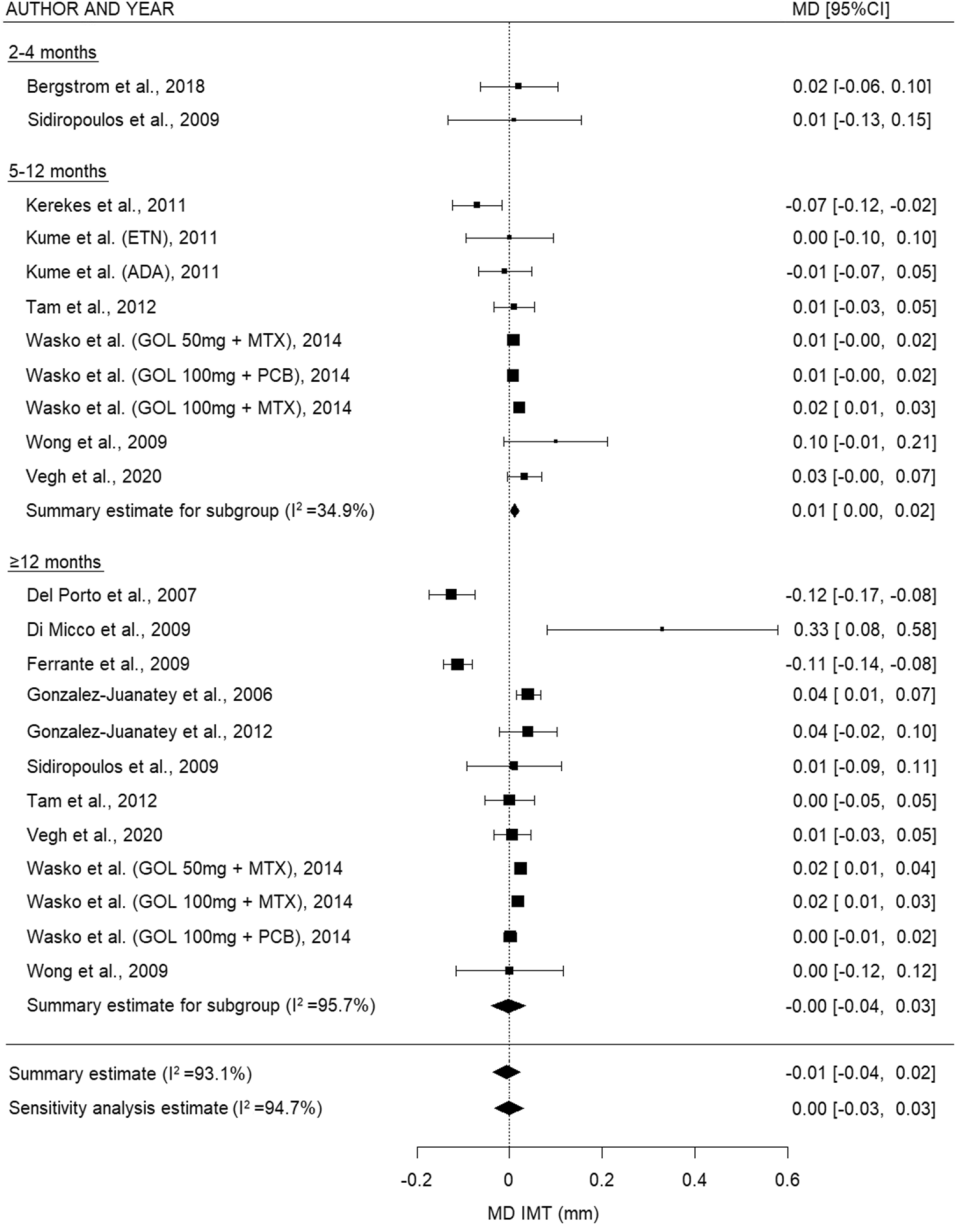
Forest plot for IMT comparing different time intervals. GOL: golimumab, IMT: intima media thickness, MD: mean difference, mm: millimeter, MTX: methotrexate, PCB: placebo, 95% CI: 95% confidence interval

Meta-analysis was performed on the 23 studies eligible for inclusion in the quantitative analysis. For PWV (Fig. 3), there was a decrease in the summary estimates of all subgroups, which was statistically significant in the overall analyses and 2–4 months (overall: −0.51 m/s (95% CI: −0.96, −0.06), *p* = 0.027, sensitivity analysis: −0.40 m/s (95% CI: −0.70, −0.11), *p* = 0.008, and 2–4 months: MD −0.77 m/s (95% CI: −1.35, −0.18), *p* = 0.011). For AIx (Fig. 4), there was a nonsignificant decrease between 2–4 and 5–12 months and in the overall analysis (2–4 months: MD −1.07% (95% CI: −2.61, 0.48), *p* = 0.177, 5–12 months: −1.79% (95% CI: −3.85, 0.26), *p* = 0.087, overall: −0.57 (95% CI: −2.11-0.96), *p* = 0.463, and sensitivity analysis: −1.21% (95% CI: −2.60, 0.19), *p* = 0.089). For IMT (Fig. 5), there was a trend toward a small increase after TNF inhibiting treatment for the relatively short- and middle-term timepoints (2–4 months: no summary statistic possible, 5–12 months: MD 0.01 mm (95% CI: 0.001, 0.02), *p* = 0.026), whereas this increase disappeared on the long-term (≥12 months: MD −0.003 mm (95% CI: −0.04, 0.03), *p* = 0.867). When all the timepoints were combined, there was no overall difference in IMT after TNF inhibiting treatment (overall: -0.01 (95% CI: −0.04, 0.02), *p* = 0.615, sensitivity analysis: −0.001 (95% CI: −0.03, 0.03), *p* = 0.947)

Heterogeneity was high between the studies with longer follow-up time (PWV ≥12 months *I*^2^=78.5%, IMT ≥12 months *I*^2^=95.7%) and the overall estimates for IMT (*I*^2^=93.1%). There was no considerably asymmetry observed in the funnel plots (Supplementary Figure S2) for PWV, which was confirmed by the results of Egger’s test (*p*=0.584). For AIx and IMT, there seemed to be some asymmetry, as more studies reported an outcome larger than the MD (AIx Egger’s test *p*=0.060; IMT Egger’s test *p*=0.134).

## Discussion

In this systematic review and meta-analysis, we aimed to provide an overview of the literature examining the effects of TNF inhibitors on PWV, AIx, and IMT in RA patients. The risk of bias in all studies with full-text had a moderate to low risk of bias, while abstracts had a high risk of bias due to a lack of information. Although the follow-up periods and the type of TNF inhibitors used in the studies varied, PWV and AIx seemed to decrease over time. IMT increased slightly in the first year of treatment, but the pooled results showed that overall IMT remained stable.

### Differences in results between studies

Differences between the effects of TNF inhibitors on the surrogate markers between the studies can be explained by several factors. First, concomitant treatments largely differed between studies and studies used different types of TNF inhibitors. Only three studies included patients with anti-TNF monotherapy, without concomitant csDMARDS [30, 33, 37]. Although all types of TNF inhibitors have been proven to be effective in RA patients, they have structural differences, which effect the binding with TNF and the stability of the complexes. It is still unclear whether these differences might result in different effects on the vascular wall of RA patients, although it has been suggested that adalimumab and etanercept might have better efficacy in decreasing PWV and AIx compared to infliximab [50, 51]. Second, the follow-up period between the studies varied in a wide range, with most studies having a relatively short follow-up period between 3 months and 1 year. On the one hand, a larger follow-up period seems to be needed to obtain unambiguous results, since the processes of arterial stiffening and atherosclerosis are slow. On the other hand, we found a large heterogeneity between the studies with longer follow-up periods, as differences in concomitant medication, differences over time in RA disease activity and other uncontrollable factors that happen over time also influence the results. In addition, other differences in baseline factors such as the presence of previous CVD, hypertension, diabetes, smoking status, and autoantibody status might have resulted in differences between study outcomes. For example, the studies of Del Porto et al. [22], Pieringer et al. [33], and Vassilopoulos et al. [39] excluded patients with previous CVD, hypertension, and/or smokers.

### Comparison with previous systematic reviews and meta-analyses

To our knowledge, this is the first systematic review and meta-analysis focusing on PWV, AIx, and IMT, specifically in RA patients. Four previous studies have investigated the body of evidence regarding the effect of TNF inhibitors on PWV, AIx, or IMT in RA or related diseases: three systematic reviews and one meta-analysis [50–53]. Two older systematic reviews of Dulai et al. [53] and Tam et al. [52], both without a meta-analysis, concluded that the balance of evidence suggests that TNF inhibitors may have beneficial effect on arterial stiffness and/or arterial wall thickness, although they state that larger more robust studies are warranted to confirm these findings. In contrast, the most recent systematic review by Knowles et al. [51], which included studies of several chronic inflammatory diseases, found that TNF inhibitors had a worsening or no effect on AIx and IMT, while their results showed a mixed effect of TNF inhibitors on PWV. The authors noticed differences between controlled and uncontrolled studies: multiple small uncontrolled studies had a positive result (a decline in surrogate markers), while larger controlled studies had a negative result (no decline in surrogate markers). In contrast, in our study, we primarily focused on the uncontrolled within-group measurements, as the control groups mostly also received anti-inflammatory therapy (e.g., methotrexate and tocilizumab) which also has an effect on the vascular wall and cardiovascular risk [16–18]. A placebo control group of RA patients without concomitant anti-inflammatory treatment would be the ideal control group to measure the effect of a TNF inhibitor on IMT, PVW, and AIx. However, it is unethical to withhold RA patients from proven effective treatment; therefore, none of the included studies had a placebo control group without concomitant anti-inflammatory treatment. Furthermore, in the study of Knowles et al., no change in the surrogate markers was considered as a negative effect [51]. Since PWV, AIx, and IMT also increase with biological age [54–57], these markers remaining stable indicate a beneficial effect of TNF inhibitors on the cardiovascular risk. Finally, our study confirms the findings of a meta-analysis of Vlachopoulos et al. [50], which only focused on PWV and AIx, included a literature search until August 2016 and also included studies with only a small amount of RA patients.

### Limitations

Our study has several limitations. The included studies varied widely in several aspects, e.g., methodology and included patients. First, we included RCTs, prospective cohort studies, and nonrandomized clinical trials. Uncontrolled cohort studies and nonrandomized trials introduce a bias toward the group with TNF inhibiting therapy. Second, the follow-up period and number of included patients differed and were in most studies small (≤ 1 year and under 50 patients), leading to high heterogeneity in some meta-analyses. Most of this heterogeneity disappeared after grouping the studies based on a follow-up period, but the studies with longer follow-up (≥12 months) still had high heterogeneity. Next to the differences between the studies that were explained above, an additional explanation might be that there was still a wide range of follow-up times in this group (12 months to 3 years). Another limitation is the lack of proper control groups and not be able to compare the mean differences of the surrogate endpoints to the group with TNF inhibitors. Only 12 studies included in the meta-analysis had a control group and the medication used in these groups varied widely. Due to insufficient data on the differences over time for IMT, PWV, and AIx for the control groups, it was not possible to compare the different anti-inflammatory treatments used in the control groups. We did not take differences in outcome measurements into consideration, such as the difference between the mean IMT of the common and internal carotid arteries, carotid-femoral PWV, and aortic PWV or the augmentation corrected for the heart rate [58, 59].

### Implications for clinical practice and future research

It has been shown that TNF inhibitors decrease the number of cardiovascular events in RA patients [60]. The mechanism behind this is largely unknown. PWV, AIx, and IMT are independent predictors of CVD [61–64] and known to be increased in RA [65–67]. This systematic review and meta-analysis is the first focusing on PWV, AIx, and IMT, specifically in RA patients and showed that IMT remained stable over time, and PWV and AIx decreased after TNF inhibiting therapy (although this was not significant for AIx). Since these markers normally increase with age, remaining stable also indicates a beneficial effect of TNF inhibitors. It is unclear whether this beneficial effect is merely due to reducing systemic inflammation, or also due to a direct effect of specifically TNF inhibitors. The exact mechanisms behind the changes of arterial stiffness and intima media thickness by TNF inhibitors need to be clarified in future research. Our results showed that effects of TNF inhibitors on surrogate markers can be measured within a short time period (within months). For future research, PWV, AIx, and IMT can be used as surrogate endpoints, especially for studies with a short (< 2 years) follow-up period, since “hard” endpoints of cardiovascular disease, e.g., cardiovascular events or mortality, are not always usable endpoints.

Proper control groups without antirheumatic drugs (other than TNF inhibitors) were not included, since withdrawing patients from effective antirheumatic treatment is considered unethical. It might however be of added value when healthy controls would be followed for the same period of time as the RA patients, to give an indication of how PWV, AIx, and IMT behave over time. In addition, clinical trials with larger sample sizes and longer follow-up times are needed, where patients preferably receive the same TNF inhibitor and concomitant treatments are similar. In our study, we did not examine the relationship between the surrogate markers and the disease activity. Studies have shown that arterial stiffness shows no significant correlation with the 28-joint Disease Activity Score (DAS28) and mixed results for IMT [68].

### Conclusion

Our systematic review and meta-analysis of 30 studies investigating the effects of TNF inhibitors on PWV, AIx, and IMT in RA patients, suggests that IMT remained stable over time, while PWV and AIx suggest a decrease. With these surrogate markers also increasing with aging, our findings indicate a favorable effect of TNF inhibitors on these well-established (surrogate) markers for subclinical cardiovascular disease and therefore on the cardiovascular disease risk. The studies showed heterogeneity considering the study design, follow-up time, and treatment patients received. Surrogate markers can be used as surrogate endpoints for CVD, especially for studies with a short follow-up period. Studies with longer follow-up periods are needed to correlate the findings with “hard” cardiovascular endpoints, taking also the phase of RA and type of TNF inhibitor into account.

## Supplementary Information

Supplementary Data S1. PRISMA Checklist (PDF 58 kb)

Supplementary Data S2. Database search strategy (XLSX 20 kb)

Supplementary Data S3. Data extraction table (XLSX 55 kb)

Supplementary Data S4. Risk of bias assessment checklist (PDF 64 kb)

Supplementary Data S5. Results risk of bias assessment (XLSX 55 kb)

Supplementary Figure S1. Overview of risk of bias assessment of abstracts (PNG 30 kb)

High Resolution Image (TIF 1368 kb)

Supplementary Figure S2. Funnel plots of IMT, PWV and AIx *P-values indicate result of Egger’s test for funnel plot asymmetry. IMT, intima media thickness; PWV, pulse wave velocity; AIx, augmentation index. (PNG 27 kb)*

High Resolution Image (TIF 799 kb)

## Data Availability

The data underlying this article are available in the article and in its online supplementary material.
