# Supplementary material for: Effect of TNF inhibitors on arterial stiffness and intima media thickness in rheumatoid arthritis: a systematic review and meta-analysis
Source: Clin Rheumatol. 2023 Jan 16;42(4):999–1011. doi: 10.1007/s10067-023-06505-y (PMC10017587; doi:10.1007/s10067-023-06505-y)
Supplement: Supplementary file 4 — Risk of bias assessment checklist (PDF 64 kb) [file 10067_2023_6505_MOESM4_ESM.pdf]

Question	yes	no	partially	unclear	not applicable
Is the hypothesis/aim/objective of the study clearly described?	1	0	0.5	0	0
Are the main outcomes to be measured clearly described in the Introduction or Methods section?	1	0	0.5	0	0
Are the characteristics of the patients included in the study clearly described?	1	0	0.5	0	0
Are the interventions of interest clearly described?	1	0	0.5	0	0
Are the distributions of principal confounders in each group of subjects to be compared clearly described?	1	0	0.5	0	0
Are the main findings of the study clearly described?	1	0	0.5	0	0
Does the study provide estimates of the random variability in the data for the main outcomes?	1	0	0.5	0	0
Have the characteristics of patients lost to follow-up been described?	1	0	0.5	0	1
Have actual probability values been reported (e.g. 0.035 rather than <0.05) for the main outcomes except where the probability value is less than 0.001?	1	0	0.5	0	0
Were the subjects asked to participate in the study representative of the entire population from which they were recruited?	1	0	0.5	0	0
Were the staff, places, and facilities where the patients were treated, representative of the treatment the majority of patients receive?	1	0	0.5	0	0
Was an attempt made to blind study subjects to the intervention they have received ?	1	0	0.5	0	0
Was an attempt made to blind those measuring the main outcomes of the intervention?	1	0	0.5	0	0
If any of the results of the study were based on “data dredging”, was this made clear?	1	0	0.5	0	1
Is the time period between the intervention and outcome the same for all patients or do the analyses adjust for different lengths of follow-up of patients?	1	0	0.5	0	0
Were the statistical tests used to assess the main outcomes appropriate?	1	0	0.5	0	0
Was compliance with the interventions reliable?	1	0	0.5	0	0
Were the main outcome measures used accurate (valid and reliable)?	1	0	0.5	0	0
For studies with within-patient comparision, was the intervention comparable for the patients?	1	0	0.5	0	0
For cohort studies and trials comparing different medications, were the patients in the different groups recruited from the same population?	1	0	0.5	0	0
Were patients recruited over the same period of time?	1	0	0.5	0	0
Were losses of patients to follow-up taken into account?	1	0	0.5	0	1
Did the study have sufficient power to detect a clinically important effect where the probability value for a difference being due to chance is less than 5%?	1	0	0.5	0	0
