# Supplementary figures and images for: Effect of TNF inhibitors on arterial stiffness and intima media thickness in rheumatoid arthritis: a systematic review and meta-analysis
Source: Clin Rheumatol. 2023 Jan 16;42(4):999–1011. doi: 10.1007/s10067-023-06505-y (PMC10017587; doi:10.1007/s10067-023-06505-y)

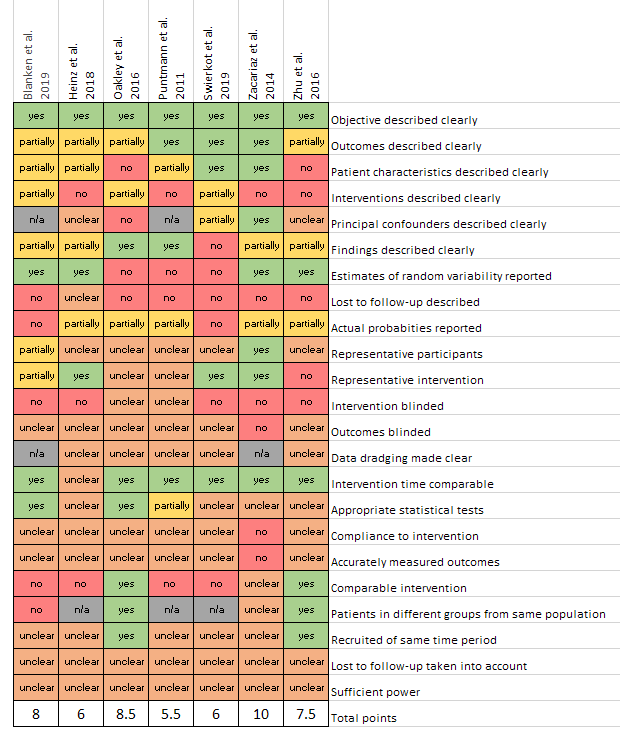

Supplement: Supplementary file 6 — Overview of risk of bias assessment of abstracts (PNG 30 kb) [file 10067_2023_6505_Fig6_ESM.png]

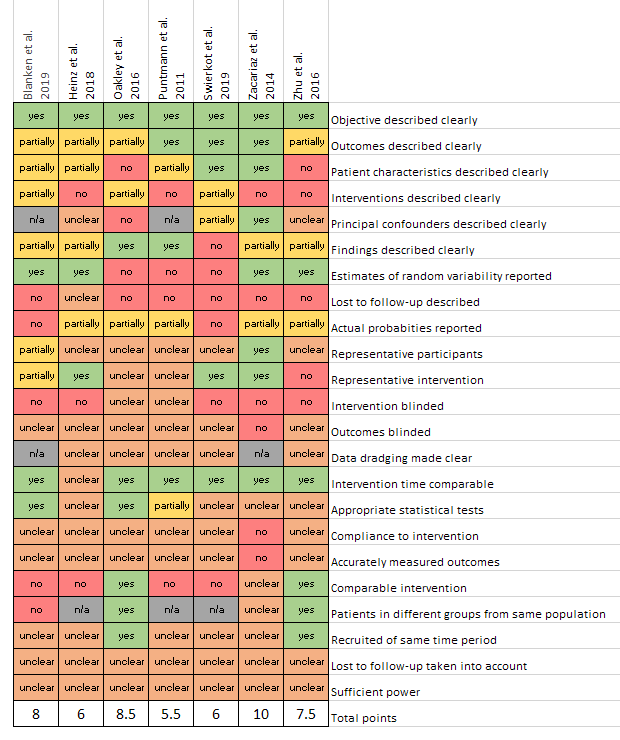

Supplement: Supplementary file 7 — High Resolution Image (TIF 1368 kb) [file 10067_2023_6505_MOESM7_ESM.tif]

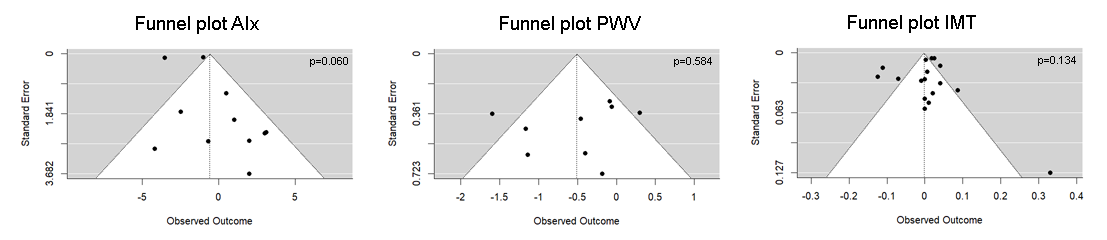

Supplement: Supplementary file 8 — Funnel plots of IMT, PWV and AIx P-values indicate result of Egger’s test for funnel plot asymmetry. IMT, intima media thickness; PWV, pulse wave velocity; AIx, augmentation index. (PNG 27 kb) [file 10067_2023_6505_Fig7_ESM.png]

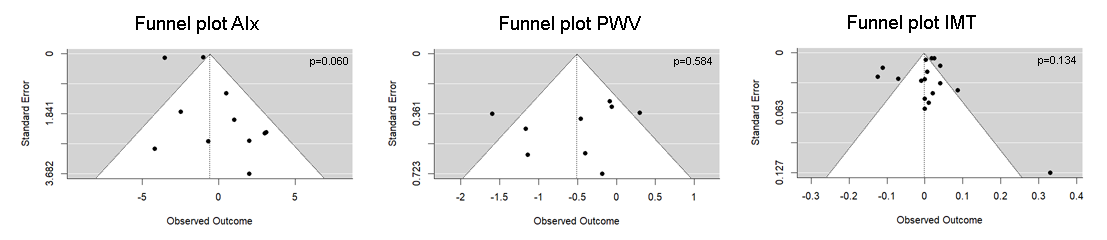

Supplement: Supplementary file 9 — High Resolution Image (TIF 799 kb) [file 10067_2023_6505_MOESM9_ESM.tif]
